# A Multi-Image Encryption Based on Sinusoidal Coding Frequency Multiplexing and Deep Learning

**DOI:** 10.3390/s21186178

**Published:** 2021-09-15

**Authors:** Qi Li, Xiangfeng Meng, Yongkai Yin, Huazheng Wu

**Affiliations:** School of Information Science and Engineering and Shandong Provincial Key Laboratory of Laser Technology and Application, Shandong University, Qingdao 266237, China; 202032785@mail.sdu.edu.cn (Q.L.); yinyongkai@sdu.edu.cn (Y.Y.); 201812554@mail.sdu.edu.cn (H.W.)

**Keywords:** optical information security, deep learning, sinusoidal coding, frequency multiplexing

## Abstract

Multi-image encryption technology is a vital branch of optical encryption technology. The traditional encryption method can only encrypt a small number of images, which greatly restricts its application in practice. In this paper, a new multi-image encryption method based on sinusoidal stripe coding frequency multiplexing and deep learning is proposed to realize the encryption of a greater number of images. In the process of encryption, several images are grouped, and each image in each group is first encoded with a random matrix and then modulated with a specific sinusoidal stripe; therefore, the dominant frequency of each group of images can be separated in the Fourier frequency domain. Each group is superimposed and scrambled to generate the final ciphertext. In the process of decryption, deep learning is used to improve the quality of decrypted image and the decryption speed. Specifically, the obtained ciphertext can be sent into the trained neural network and then the plaintext image can be reconstructed directly. Experimental analysis shows that when 32 images are encrypted, the CC of the decrypted result can reach more than 0.99. The efficiency of the proposed encryption method is proved in terms of histogram analysis, adjacent pixels correlation analysis, anti-noise attack analysis and resistance to occlusion attacks analysis. The encryption method has the advantages of large amount of information, good robustness and fast decryption speed.

## 1. Introduction

As the development of the Internet, networks and information systems are playing an increasingly important role in people’s life, work and study. However, as people’s expectations for informatization have deepened, a subject that cannot be ignored has been placed in front of people, that is, the security of information. The resolution of these security issues depends on the progress and development of information security technology. Therefore, the research on information security technology plays a vital role, which not only has academic value but also plays an important role in promoting the development of the entire human society. Data encryption technology based on optical theory and method is a new generation of information security theory and technology that has begun to develop internationally in recent years. Compared with traditional information security technology, optical information security technology has the following advantages: firstly, optical cryptography system has parallelism [1]. Secondly, optical cryptography systems usually have large key space. Thirdly, optical cryptography system has the characteristics of multi-dimension [2]. Some inherent parameters of the system, such as amplitude, phase, wavelength and optical element parameters, can be used as the key parameters of the optical cryptosystem to achieve multi-dimensional encryption. Therefore, optical information security technology has the characteristics of large capacity, fast storage speed, multi-dimensional parallel processing and so on and has unique advantages in data transmission and protection. Today, with the growing development of optical information technology, optical image encryption technology has made great progress.

In 1995, Refregier et al. [3] proposed an optical image encryption scheme based on double random phase encoding for the first time, which means it uses two uncorrelated random phase templates and Fourier transform to realize optical image encryption. In essence, this scheme is based on optical transformation to disturb the information of plaintext images and generate ciphertext images, such as fractional Fourier transform [4], fractional wavelet transform [5], fractional Merlin transform [6], interference [7], single-pixel imaging [8,9], etc. Optical image encryption belongs to parallel encryption with high speed and high efficiency, but its encryption performance is limited by the technology and precision of various optical devices in the optical path [10].

In addition, many previous studies [11,12,13,14,15] only consider the encryption of a single image, which greatly reduced the efficiency of the encryption system. As another important branch of optical encryption technology, multi-image encryption technology has attracted an increasing number of attention since it not only improves the encryption ability but also reduces the amount of ciphertext data. In recent years, multiple image encryption methods have been proposed based on wavelength multiplexing [16], multiplexing position [17], phase mask only (PMO) multiplexing [18], lateral transfer [19], optical data compression [20] and so on. Lee and Cho [21] proposed a double random phase encryption method for multi-image transmission based on orthogonal coding. Li et al. [22] proposed a multi-image encryption method based on compressed ghost imaging. Wu et al. [23] proposed a multi-image encryption scheme based on different diffraction distances to calculate ghost images. Zhang et al. [24] proposed a multi-image encryption scheme based on the concept of compressed ghost imaging and the sampling principle of Fourier transform. Yang [25] et al. proposed a multi-image encryption scheme based on compression coding aperture imaging. However, with the advent of the era of big data, a growing number of data need to be transmitted, and the above encryption methods can only encrypt a small number of images. At the same time, during decryption, it takes a lot more time than using the traditional multi-image encryption method, which greatly limits its application in practice. This inspired us to adopt a more efficient method to encrypt more images.

The energy of a natural image in the Fourier frequency domain is concentrated in the low-frequency component [26,27], and such multiplexing of the frequency information would be of few resolution loss. Codes with Fourier coefficients following impulse-shaped distribution can conduct effective frequency multiplexing [28]. Typical Fourier encoding is sinusoidal modulation, which shifts the Fourier spectrum of the original image to a specific region determined by the modulation frequency.

Inspired by the above theory, this paper introduces the sinusoidal stripe coding on the basis of random matrix. Specifically, the plaintext images are grouped before encryption, and each group of images is first subjected to random matrix coding and then sinusoidal stripe coding during encryption. The specially designed sinusoidal stripe moves the frequency components of each group of images in the Fourier frequency domain with different offsets. In this way, the number of encrypted images is far more than that of the traditional encryption method. In order to improve the quality of decrypted image and the speed of decryption, deep learning (DL) is used to reconstruct the plaintext during decryption, which is a powerful tool in many areas [29,30,31,32,33,34]. A predominant characteristic of DL is that it enables neural networks to automatically analyze the relationship between data and data. Therefore, we can use this characteristic of the neural network to study the corresponding relationship between the plaintext and the ciphertext in optical image encryption, that is to say, we can successfully realize the ciphertext decryption by analyzing the relationship between them.

The main contributions of the proposed encryption methods can be summarized as follows:

1. A multi-image encryption method based on sinusoidal coding frequency multiplexing and deep learning is proposed.
2. The proposed encryption method can realize the encryption of a greater number of images, which makes it more widely used.
3. In the process of decryption, deep learning is used to improve the quality of the decrypted image and the decryption speed.

The rest of this paper is organized as follows. The second part is to analyze the theories, which will include the introduction of sinusoidal stripes and the adoption of deep neural networks. The third part will explore the process of encryption and decryption in detail. Furthermore, the security and robustness will be analyzed in the fourth part. The final part is a conclusion.

## 2. The Theoretical Analysis

### 2.1. The Encryption Process

**Figure 1:**
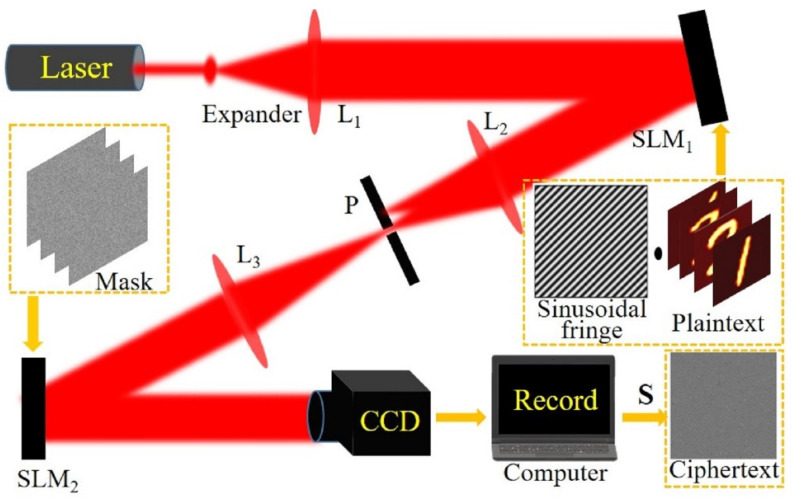
Schematic diagram of multi-image encryption and coding scheme.

A multiplexed coding scheme will be proposed to encrypt images, and the encryption process is shown in Figure 1.

Assuming that there are many *n* × *m* plaintext images, the encryption process is described as follows:

1. All the *n* × *m* plaintext images are divided into n groups; the plaintext images in each group and the sinusoidal code corresponding to each group of the plaintext images are successively sent to a spatial light modulator (SLM1) for display.
2. The L2 and L3 lenses form the 4F system, and the hole P is located on the spectral plane of the 4F system, which is used to extract the zero-order frequency after SLM1 and reduce the influence of errors caused by other orders. The random matrix corresponding to each plaintext image is uploaded to SLM2 for coding.
3. The superimposed light intensity recorded by the CCD is the time integral of the light field reaching the target surface within a certain period of time. Therefore, we make the exposure time of the CCD equal to the sum of the encoding time of all images in the SLM. In addition, their starting time should be synchronized, and the encoding time of each image should also be the same, which can be expressed as follows:

(1) $$I(\vec{p})={\sum_{j=1}^{n}\sum_{i=1}^{m}s_{j}(\vec{p})}_{}^{}r_{i}^{}{\left(\vec{p}\right)}_{}^{}I_{ij}^{}(\vec{p})$$

Here $I(\vec{p})$ represents the ciphertext, $s_{j}(\vec{p})$ represents the *j*-th specially designed sinusoidal stripe, $r_{i}^{}(\vec{p})$ represents the *i*-th random matrix, $I_{ij}^{}(\vec{p})$ represents the plaintext image, and ∑(·) represents the sum of the elements.

1. According to the number of pixels with superimposed light intensity, an integer random sequence without repeating elements is generated. Secondly, replace the light intensity value on each pixel of the superimposed light intensity according to the value of the random sequence, so as to realize the scrambling operation. Scramble (S) [25] $I(\vec{p})$ to get the final ciphertext.

### 2.2. The Decryption Process

#### 2.2.1. Downsampling in Fourier Frequency Domain

**Figure 2:**
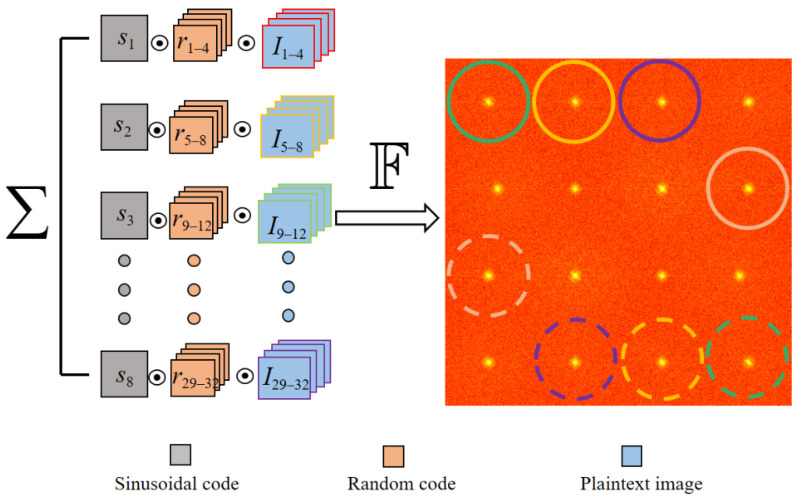
The coding scheme and the Fourier spectrum.

If we convert the obtained ciphertext image to the Fourier domain after scrambling recovery, its spectrum can be expressed as:

(2) $$\tilde{I}(\vec{p})=\sum_{j=1}^{n}\sum_{i=1}^{m}\left[a\delta (\vec{\omega })+\frac{b}{2}(\vec{\omega }+{\vec{\omega }}_{j})e^{-\phi }+\frac{b}{2}(\vec{\omega }-{\vec{\omega }}_{j})e^{\phi }\right]*F\left[r_{i}^{}(\vec{p})I_{ij}^{}(\vec{p})\right]$$

where F[•] represents the Fourier transform, $\vec{\omega }$ and ϕ represent the frequency and phase of the sinusoidal stripe, respectively. As shown in Figure 2, here we take *m* = 4 and *n* = 8 as an example. Each group is coded using four random matrices, followed by eight sinusoidal codes that move the frequency components of each group by different offsets; therefore, their dominant frequencies are staggered in Fourier domain. For each group, the shifted frequencies have two symmetrically conjugated positions (denoted by solid and dashed circular of the same color).

Since most energy of the plaintext images and the random matrix concentrates at low frequencies, $\sum_{i=1}^{m}r_{i}^{}(\vec{p})I_{ij}^{}(\vec{p})$ can be extracted via the operation ε as follows:

(3) $$I_{j}^{}(\vec{p})=\sum_{i=1}^{m}r_{i}^{}(\vec{p})I_{ij}^{}(\vec{p})=\frac{2}{b}\left|F^{-1}\left[\varepsilon \left[\tilde{I}(\vec{p})\right]\right]\right|$$

Here the operation ε includes two steps: first extracting the Fourier modulus from $\tilde{I}(\vec{p})$, and then padding its surroundings with zeros to keep the original pixel resolution.

Then, *I_j_* is taken as the input to the network, and the output is *m* plaintext images. The detailed process of deep neural network decryption will be described in the next section. So far, we can extract and decrypt *m* plaintext images from each group of *I_j_*.

#### 2.2.2. The Network Structure

**Figure 3:**
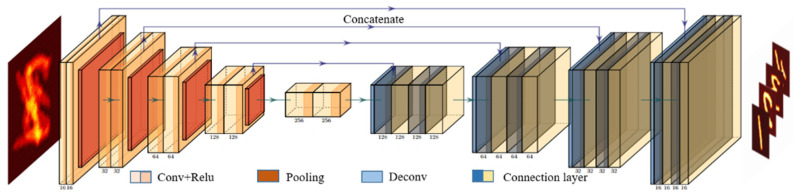
U-Net architecture.

Deep learning (DL) is a powerful tool in many areas. In the aspect of network structure, this paper adopts the classic U-Net [35] network structure, which is applied to plaintext reconstruction after modifying the output layer and loss function of U-Net. The input of the network is a single-channel two-dimensional image, and the final output is an m-channel two-dimensional image after passing through five down-sampling convolutional layers and five up-sampling deconvolution layers. Each channel represents a plaintext image. Its network structure and specific parameters of each layer are shown in Figure 3.

In the process of neural network training, the datasets play an important role. A high quality dataset can often improve the quality of model training, speed up the progress of training and improve the final output results. Considering the particularity of the image encryption method proposed in this paper, we choose the self-made dataset, in which the data pair consists of m plaintext images and ciphertext. In this paper, 15,000 images are selected from MNIST handwritten dataset as the plaintext images, and then encrypted according to the encryption method mentioned above, so as to obtain the ciphertext-plaintext data pairs.

In order to better restore the plaintext images, mean-square error (MSE) is utilized and defined as follows:

(4) $$MSE=\frac{1}{M\times N}\sum_{i=1}^{M\times N}{\left(x_{i}-y_{i}\right)}^{2}$$

where *M* and *N* represent the width and height of the image respectively, and $x_{i}$ and $y_{i}$ represent the output value of the last layer of the network and the truth value of the original image respectively. Since the last layer of the modified U-Net network is *M*-channel, the loss function is:

(5) $$Loss_{1}=\frac{1}{M\times N}\sum_{i=1}^{m}\sum_{j=1}^{M\times N}{\left(x_{ij}-y_{ij}\right)}_{}^{2}$$

We further optimize the loss function and the benefit of this is to improve the training ability of the network. Specifically, the m plaintext images output from the network are encrypted again according to the proposed encryption method, and then the *MSE* is calculated with the real ciphertext, which can be expressed as:

(6) $$Loss_{2}=MSE(I(\vec{p}),I^{’}(\vec{p}))=\frac{1}{M\times N}\sum_{i=1}^{M\times N}{\left(I_{i}(\vec{p})-I_{i}{}^{’}(\vec{p})\right)}^{2}$$

where $I(\vec{p})$ represents the real ciphertext, and $I^{’}(\vec{p})$ represents the reconstruction ciphertext that is re-encrypted using the plaintext image output from the network. Therefore, the total loss function is:

(7) $$Loss=Loss_{1}+Losss_{2}=\frac{1}{M\times N}\sum_{i=1}^{m}\sum_{j=1}^{M\times N}{\left(x_{ij}-y_{ij}\right)}^{2}+\frac{1}{M\times N}\sum_{i=1}^{M\times N}{\left(I_{i}(\vec{p})-I_{i}{}^{’}(\vec{p})\right)}^{2}$$

In the training process, the learning rate is set to 0.001 and the Adam optimizer [36] is used to optimize and update the parameters of the network. The number of training epoch is 50. All programs run in Python 3.7 with NVIDIA GeForce GTX 3060 GPU for acceleration.

## 3. Experiment Results

**Figure 4:**
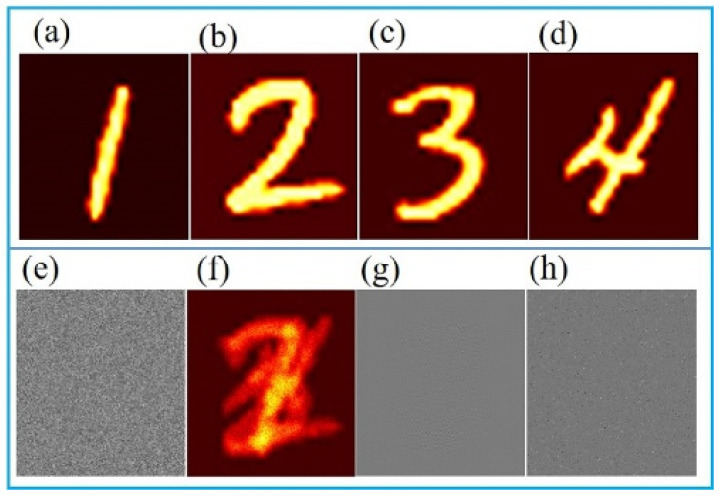
(**a**–**d**) Four plaintext images in the first group. (**e**) One of the random matrices. (**f**) The group ciphertext. (**g**) After superposition ciphertext. (**h**) After scrambling ciphertext.

In order to prove the feasibility of our method in encryption and the superiority of decryption, we conduct numerical simulation experiments to verify it. In the process of encryption, 32 images are selected from MNIST handwritten digital dataset and divided into eight groups, with four images in each group, and the resolution is 256 × 256. Figure 4 shows the encryption process for multiple images.

The first line (a–d) in Figure 4 shows the plaintext images in the first group, Figure 4e represents one of the corresponding four random matrices, and Figure 4f–h represent the images encoded by random codes and sinusoidal stripes of the first group, the 8 groups of superimposed ciphertext image and the scrambled ciphertext images respectively. It can be found intuitively that it is almost impossible to detect any information of the original image from the ciphertext.

**Figure 5:**
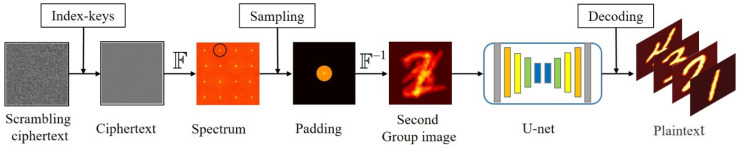
The decryption process.

As shown in Figure 5, the detailed decryption process is described as follows:

1. The pixel location of the ciphertext is rearranged by the correct index keys to get the superimposed images;
2. Fourier transform is applied to the superimposed ciphertext image and appropriate down-sampling is carried out according to the specific spectrum distribution of each group;
3. Its surroundings are padded with zeros to keep the original pixel resolution;
4. Inverse Fourier transform is carried out and it is fed into the trained U-Net network.

**Figure 6:**
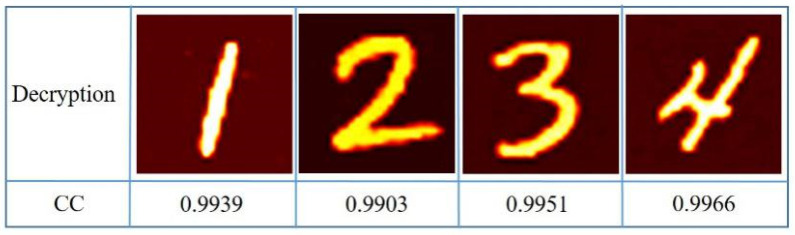
The first set of decryption results.

The correlation coefficient (CC) is used to calculate the similarity between the original plaintext image and the decrypted image, which is defined as follows:

(8) $$CC=\frac{E\left\{\left[I_{t}-E(I_{t})\right]-[I-E(I)]\right\}}{\sigma_{I_{t}}\sigma_{I}}$$

where E{·} denotes the expected value operator, and σ is the standard deviation of the corresponding image. *I_t_* and *I* are the original plaintext images and decrypted images, respectively. The closer CC value is to 1.0, the better the quality of the reconstructed images. The decryption results are shown in Figure 6, with CC of 0.9939, 0.9903, 0.9951, and 0.9966, respectively.

**Figure 7:**
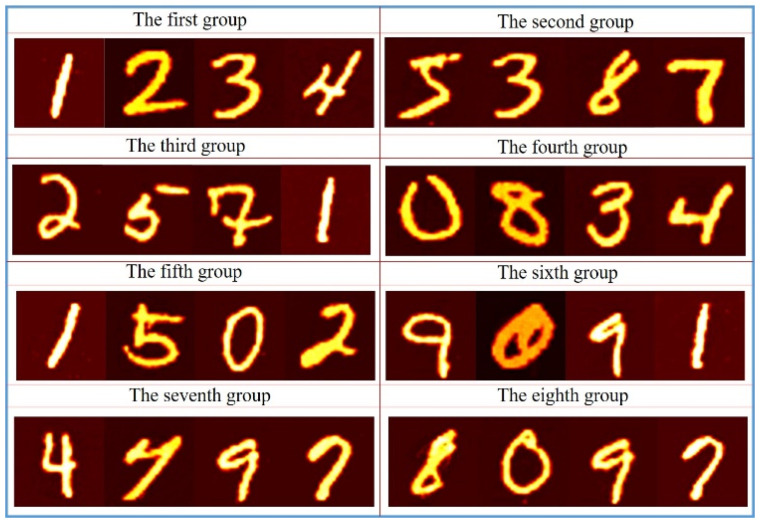
Decryption results for all groups.

Obviously, these final decrypted images with high quality are very similar to the corresponding plaintext images. At the same time, as the deep neural network is used to decrypt, the decryption time is very fast. Using Intel(R) Core(TM) i7-9700K CPU without using GPU acceleration, the entire decryption process can be completed in only 3.15 s. The decryption results for all groups are shown in Figure 7.

**Figure 8:**
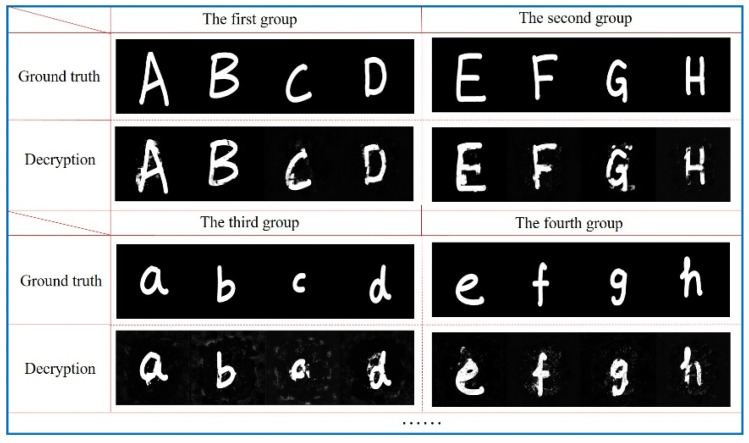
Testing results for handwritten English alphabet.

In addition, we use some plaintext images that do not belong to the training set for reconstruction to test the generalization of the encryption model proposed in this paper. For convenience, Handwritten English alphabets are used for testing and the reconstructed plaintext images are shown in Figure 8.

Although the network has been trained with MNIST handwritten datasets, it can perform high-quality reconstruction of handwritten English alphabet, which indicates that the U-Net networks can learn the correspondence between ciphertext images and original plaintext images very well.

**Figure 9:**
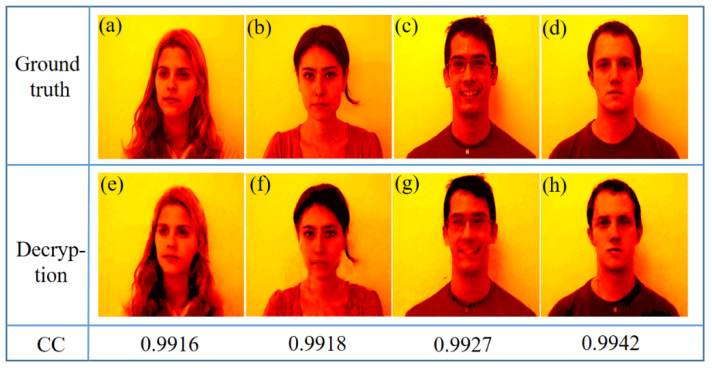
FEI face decryption results. (**a**–**d**) Groudtruth; (**e**–**h**) Decryption.

In order to further verify the feasibility of the encryption method, we use FEI FACE Database [37] for training, in which the images are gray images with more complex content and richer details. In the process of encryption, four face images are used and divided into two groups, according to the encryption steps mentioned above. By reducing the number of encrypted images, each group of images can obtain a large sampling rate in the Fourier frequency domain to ensure high quality plaintext reconstruction. The decryption result is shown in Figure 9.

## 4. Algorithm Analysis

We know that the ciphertext and the key will inevitably be attacked or changed in the process of transmission, such as data missing, affected by the noise and so on. A good information security system can not only guarantee the confidentiality of information but also ensure the integrity of information decryption, so the information encryption system proposed by researchers is required to have good security and robustness.

### 4.1. Key Security Analysis

**Figure 10:**
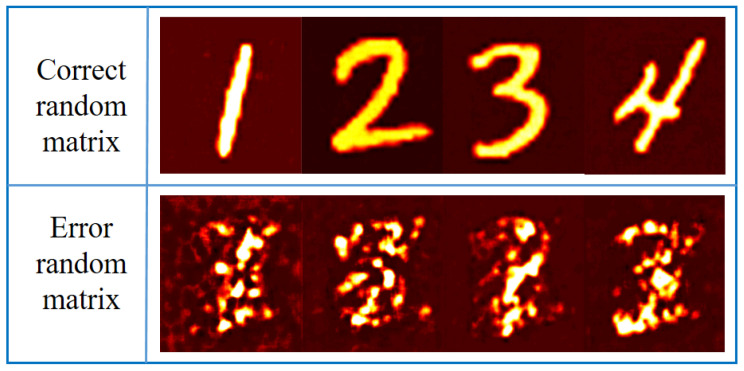
Decryption results under different random matrices.

The proposed encryption method has great security, even if the attacker knows what kind of network structure to use and the corresponding encryption method, and tries to attack the encryption system through training, but the use of random matrix in the encryption process is unknown, based on the wrong random matrix training network leading to decryption failure. The top row of Figure 10 shows the decryption results under the correct random matrix, and the bottom row shows four images that failed to decrypt based on incorrect random matrix training.

It can be seen that if the wrong random matrix is used for training, no useful information can be seen from the decryption results. This fully shows that the proposed encryption algorithm has good security.

### 4.2. Anti-Noise Attack Analysis

**Figure 11:**
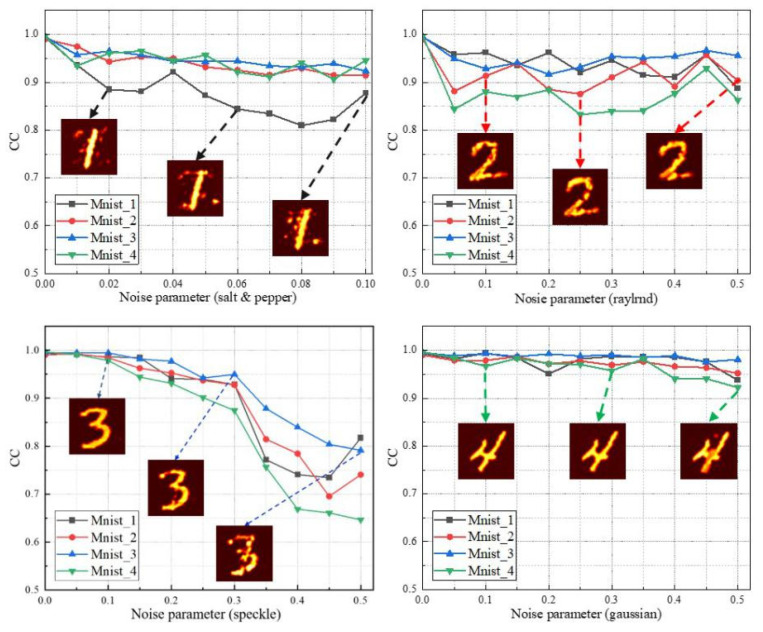
The CC curve of the decryption results after adding different noise attacks to the ciphertext.

It is also necessary to evaluate the robustness of the encryption algorithm when the ciphertext is attacked by noise. In order to verify that the encryption method has good anti-noise ability, speckle noise and Gaussian noise are added in the process of network training to improve the robustness. The mean values of speckle noise and Gaussian noise added in the training process are all 0, and the variance is randomly selected in (0.05, 0.06, 0.07, 0.08, 0.1). At the same time, four different types of noise attacks are added into the ciphertext: Gaussian noise, speckle noise, raylrnd noise and salt and pepper noise. CC change curve of decrypted image under noise attack is shown in Figure 11.

For Gaussian noise and speckle noise, the abscissa represents the variance parameter. It can be seen from Figure 11 that the proposed encryption model has a very good ability to resist Gaussian noise. Even when the variance is 0.5, CC is still higher than 0.9. At the same time, when the network is attacked by other kinds of noise, although the decryption quality decreases with the increase of noise, the image can still be decrypted clearly.

### 4.3. Resistance to Occlusion Attacks

**Figure 12:**
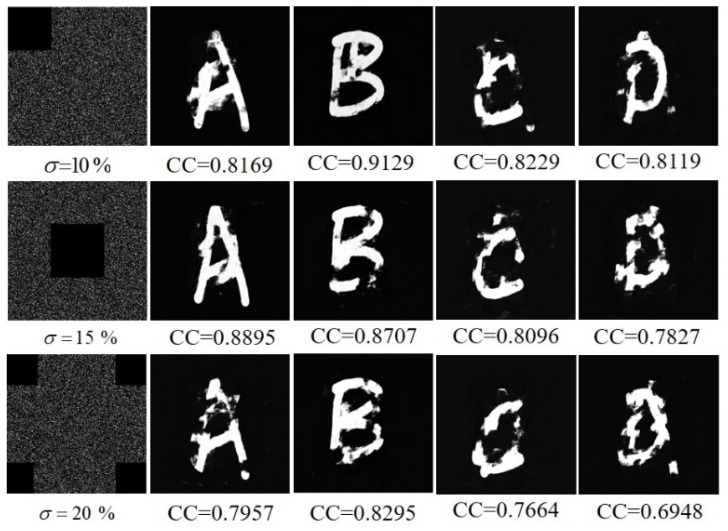
Decryption results after different degrees of occlusion attacks on ciphertext.

Next, we analyze the influence of the occlusion attack on the decryption results. Three different occlusion styles and their corresponding decrypted images are shown in Figure 12. The corresponding plaintext images are shown on the right side of Figure 12, from which the primary information of the original plaintext images can be recognized visually.

### 4.4. Correlation Analysis

The correlation of adjacent pixels reflects the correlation degree of pixel values at adjacent positions of the image. A secure encryption algorithm should reduce the degree of correlation between pixel values in adjacent positions of the image. The correlation of the image should include horizontal correlation, vertical correlation and diagonal correlation. The formula for calculating correlation coefficient is as follows:

(9) $$r_{xy}=\frac{\mathrm{cov}(x,y)}{\sqrt{D(x)D(y)}}$$

(10) $$E(x)=\frac{1}{N}\times \sum_{i=1}^{N}x_{i},{}_{}D(x)=\frac{1}{N}\times \sum_{i=1}^{N}{\left(x_{i}-E(x)\right)}^{2}$$

(11) $$\mathrm{cov}(x,y)=\frac{1}{N}\times \sum_{i=1}^{N}\left(x_{i}-E(x))(y_{i}-E(y)\right)$$

As shown Table 1, the correlation coefficients between adjacent pixels of plaintext images are all greater than 0.9, indicating a high correlation. In the ciphertext image, the average correlation coefficient of adjacent pixels in three directions is closer to 0. This means that the pixel distribution of the ciphertext image is very chaotic and there is no statistical correlation.

**Figure 13:**
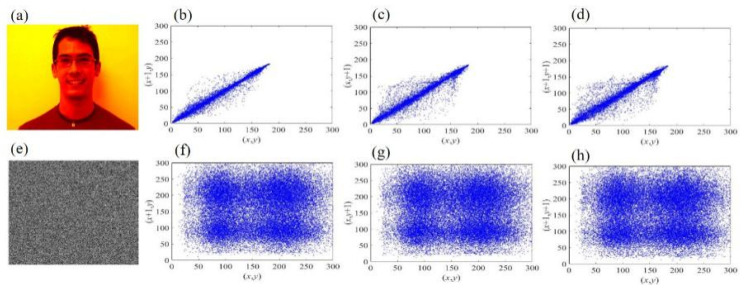
(**a**) One of the plaintext images. Correlation distributions of the plaintext image in horizontal (**b**), vertical (**c**) and diagonal (**d**), respectively. (**e**) Ciphertext image. Correlation distributions of the ciphertext image in horizontal (**f**), vertical (**g**) and diagonal (**h**), respectively.

At the same time, in order to show the correlation between adjacent pixels of the image intuitively, one of the four plaintext images is selected and the correlation analysis diagram of three directions is drawn. The correlation analysis results are shown in Figure 13.

The experimental results show that the adjacent pixels of the ciphertext image have low correlation in horizontal, vertical and diagonal directions, which reduces the statistical characteristics of pixel correlation, thus proving that the proposed method for image encryption can resist the statistical attack based on pixel correlation.

### 4.5. Histogram Analysis

**Figure 14:**
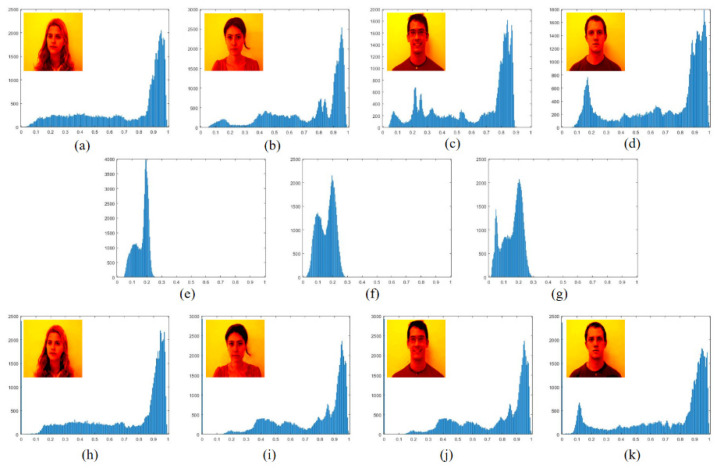
(**a**–**d**) Histograms of 4 plaintext images. (**e**) Superimposed light intensity histogram after encoding the first two images. (**f**) Superimposed light intensity histogram after encoding of the last two images. (**g**) Histogram of the final ciphertext image. (**h**–**k**) Histograms of the decrypted image.

In the case of image feature leakage, it is vital that the encryption scheme can resist statistical analysis. The histogram of the image shows the distribution of pixel values in the image, so the histogram is a key indicator reflecting the robustness of the image encryption scheme [38]. The histograms of four plaintext images, ciphertext images, and decrypted images are shown in Figure 14. The experimental results show that the distribution of pixel values in the ciphertext image is significantly different from that of the plaintext image. It can be seen from the histograms of the four plaintext images that the encryption model has successfully changed the distribution of pixel values, removed the statistical characteristics of pixel values, and can effectively resist attacks based on statistical analysis.

### 4.6. Analysis of the Number of Encrypted Images

**Figure 15:**
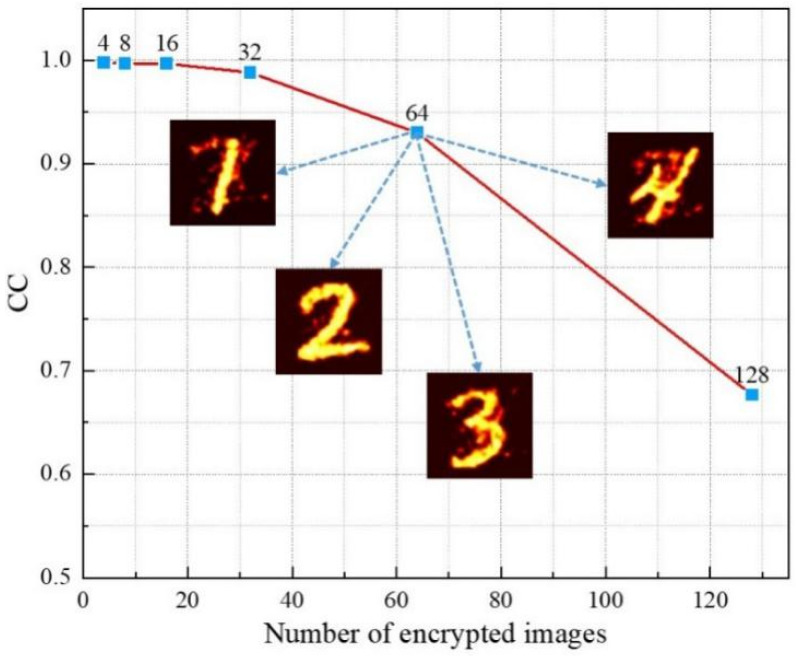
The relationship between the number of encrypted images and the quality of decrypted images.

The number of encrypted images in multiple image encryption will directly affect the application of the algorithm in practice. Next, we analyze the relationship between the number of encrypted images and the quality of decrypted images. As shown in Figure 15, taking four images per group, as the number of encrypted images increases, the decryption result decreases. However, when the number of encrypted images is 64, the content of the image can still be clearly distinguished, which cannot be achieved by the traditional multi-image encryption algorithm [21,22,23,24,25]. Due to the limitation of image size, the sampling ratio in the frequency domain is too small to achieve better decryption when the number of encrypted images is 128.

## 5. Conclusions

In this paper, we propose a multi-image encryption method based on deep learning and sinusoidal stripe coding frequency multiplexing. The CCD camera can detect the superposed image after grouping, random matrix coding and sinusoidal stripe modulation operation. Then the deep neural network is trained to learn the correspondence between the plaintext and the ciphertext. After the training, the ciphertext image is transmitted to the trained network for decryption after scrambling recovery, Fourier transform, downsampling and inverse Fourier transform. Compared with the previous multi-image encryption methods, the proposed encryption method has more encrypted images and faster decryption speed, which makes it more widely used. Moreover, theoretical analysis, numerical simulation experiment results and robustness test all verify the feasibility and safety of the proposed method. In future work, we will further optimize the encryption method and deep neural network structure to enable it to encrypt more general grayscale images. Furthermore, the Bayer matrix can be used to preprocess color images into grayscale images, which is expected to restore the original color of the decrypted image after the introduction of De-Mosaic algorithm, thereby realizing color image encryption.

## Figures and Tables

**Table 1:** Correlation coefficient between adjacent pixels of plaintext image and ciphertext image in each of three directions.

Test Image	Horizontal	Vertical	Diagonal
Img1	0.9831	0.9749	0.9779
Img2	0.9689	0.9594	0.9638
Img3	0.9337	0.9220	0.9321
Img4	0.9605	0.9487	0.9548
Ciphertext	−0.0115	−0.0063	0.0038

## Data Availability

Publicly available datasets were analyzed in this study. This data can be found here: https://github.com/liqi-sdu/Test (accessed on 14 September 2021).
